# Study of Generalized Chaotic Synchronization Method Incorporating Error-Feedback Coefficients

**DOI:** 10.3390/e25050818

**Published:** 2023-05-18

**Authors:** Yanan Xing, Wenjie Dong, Jian Zeng, Pengteng Guo, Jing Zhang, Qun Ding

**Affiliations:** 1 Electronic Engineering College, Heilongjiang University, Harbin 150080, China; yananxinghlj@aliyun.com (Y.X.); 2211790@s.hlju.edu.cn (J.Z.);; 2 Information Engineering College, Heilongjiang Polytechnic, Harbin 150080, China; 3 Beijing Aerospace Institute of Automatic Control, Beijing 100854, China

**Keywords:** chaotic synchronization, generalized synchronization, chaotic hiding and anti-hiding, parameter control, transmission system

## Abstract

In this paper, taking the generalized synchronization problem of discrete chaotic systems as a starting point, a generalized synchronization method incorporating error-feedback coefficients into the controller based on the generalized chaos synchronization theory and stability theorem for nonlinear systems is proposed. Two discrete chaotic systems with different dimensions are constructed in this paper, the dynamics of the proposed systems are analyzed, and finally, the phase diagrams, Lyapunov exponent diagrams, and bifurcation diagrams of these are shown and described. The experimental results show that the design of the adaptive generalized synchronization system is achievable in cases in which the error-feedback coefficient satisfies certain conditions. Finally, a chaotic hiding image encryption transmission system based on a generalized synchronization approach is proposed, in which an error-feedback coefficient is introduced into the controller.

## 1. Introduction

Chaos is a unique nonlinear dynamical phenomenon with the properties of ergodicity, initial sensitivity, and the long-term unpredictability of motion trajectories [1,2,3,4]. In recent years, the study of chaos has become very popular, and it is widely used in the field of secure communication [5,6,7]. Chaos control and synchronization theory, which has great potential for application in the field of chaos research, has also become a hot spot in the high-tech competition between countries [8,9]. From the point of view of chaotic system interactions, studies related to chaotic synchronization can be divided into the following categories: generalized synchronization, phase synchronization, hysteresis synchronization, and so on [10,11,12,13]. In addition, during the process of research, researchers have proposed complete synchronization, projective synchronization, and adaptive synchronization [14,15,16,17]. In practical studies, the problem of parameter selection is inevitable regarding the structural differences between the drive and response systems. The generalized synchronization problem for chaotic or hyperchaotic systems would be a more relevant and worthwhile approach, given that the problems mentioned above can be easily solved for generalized chaotic synchronization systems. Meanwhile, the development of generalized synchronization theory has provided new tools for constructing more secure communication systems.

Generalized synchronization is the gradual convergence of the trajectory curves of two chaotic systems to a time-independent transformation relationship over time; that is, a functional relationship is determined between the state of the driven system and the state of the responding system, and the synchronization of the driven and responding systems is achieved by this functional relationship, which can be deterministic or nondeterministic [18,19,20,21,22]. This paper proposes a generalized chaotic synchronization method incorporating error-feedback coefficients into the process of determining the function relationships, which is based on the principle of using the relationships between the functions in the designed controller to synchronize the drive and response systems. Most of the systems used in practical engineering are high-dimensional nonlinear systems. With the continuous research in applied mathematical theory and the rapid development of computer technology, lower-dimensional chaotic systems in practical applications are facing more challenges; hence, high-dimensional hyperchaos with more than two Lyapunov exponents is of significant interest. Based on the above, new 3D and 6D discrete chaotic systems are constructed and proposed in this paper. The constructed new high-dimensional chaotic systems are used as the driving systems, and the response system is constructed by the proposed generalized chaotic synchronization method that incorporates error-feedback coefficients. The effectiveness of the synchronization method was confirmed by experiment.

The paper is organized as follows: The generalized synchronization theoretic of discrete chaotic systems and the stability principle of error systems are analyzed in Section 2. A new 3D discrete chaotic system is proposed in Section 3, in which the dynamic behavior of phase diagrams, Lyapunov exponent diagrams, and bifurcation diagrams are depicted and analyzed. Subsequently, a generalized chaotic synchronization method incorporating error-feedback coefficients is proposed, with a new 3D discrete chaotic system as the driving system. The effectiveness of the method was verified by experimental simulations. In Section 4, a new 6D discrete chaotic system is proposed, and the dynamic behavioral properties of its phase diagram, Lyapunov exponent diagram, and bifurcation diagram are analyzed. Then, the new 6D discrete chaotic system is applied as the driving system through the proposed generalized chaotic synchronization method incorporating error-feedback coefficients; the effectiveness of the method was further demonstrated by performing simulations. In Section 5, a digital image transmission system based on 6D chaotic synchronization and encryption is proposed, the encryption and decryption processes are analyzed in detail, and encryption and decryption simulations are given. Then, in Section 6, security analyses are carried out based on the previously proposed encrypted image transmission system. Finally, the conclusion is given in the last section.

## 2. Theory of Generalized Synchronization for Discrete Chaotic Systems

In our study of chaotic control problems, it is more important to convert the problem of chaotic synchronization into the analysis of system errors. The main idea is to consider the difference in the state between the drive and response systems, that is, the synchronization error of the system. Once a reasonable controller has been designed by parameter changes to make the system error asymptotically stable at the origin point, then the two systems can be considered synchronized with each other. Firstly, the mathematical model of generalized chaotic synchronization is proposed in this paper and described, as follows.

**Definition** **1.** *Consider two n-dimensional nonlinear dynamical systems, and describe them using the following equations:*

(1) X(k+1)=F(X(k))

(2) Y(k+1)=Q(Y(k))+G(X(k),Y(k))

*where* X*,* Y∈ℝ*, and* F(⋅) *as well as* Q(⋅) *are n-dimensional nonlinear functions, and* G(⋅) *is an n-dimensional input control function. If the selectable function* G(X(k), Y(k)) *is applied such that* k→∞*, and thus* $\underset{k\to \infty }{\lim}\left\|G\left(X\left(k\right),\;Y\left(k\right)\right)\right\|=0$*, then it can be translated into the study of the error system *(e(k))*, for which* e(k)=G(X(k), Y(k))*, and therefore* $\underset{k\to \infty }{\lim}\left\|e\left(k\right)\right\|=0$*. In this case, the drive system and response system can reach a generalized synchronization.*

**Theorem** **1.** *Define an invertible transform* *(*$H:{\mathbb{R}}^{m}\to {\mathbb{R}}^{m}$*);consequently, there is an incorporated error-feedback coefficient *(η)*.Where the feedback coefficient satisfies the condition* η∈(−1,1)*, it can enable the progressive stability of the zero solution of Error Equation (3) of the system, which is represented as follows:*

(3) $$e\left(k+1\right)=H\left(X_{m}\left(k+1\right)\right)-Y\left(k+1\right)$$

Because the zero solution of Equation (3) is gradually stable, by introducing a reasonable feedback coefficient (η), the drive and response systems can be synchronized in a universal way.

Hence, according to **Theorem 1**, it can be concluded that to synchronize the drive system (1) and response system (2), a nonlinear error system (e(k)) needs to be constructed, and the progressive stability of the error equation of the system ($e\left(k+1\right)=H\left(X_{m}\left(k+1\right)\right)-Y\left(k+1\right)$) needs to be guaranteed. Based on the above, the next major concern is to determine that the system error equation is asymptotically stable at the original point; therefore, the following lemmas are given:

**Lemma** **1** ([23])**.** *Given a linear discrete system, which can be defined as follows:*

(4) x(k+1)=Ax(k)

*where A is a* n×n *coefficient matrix, and* $A\in R^{n\times n}$*, we can draw the following conclusions:*

*(1)*  *Chaotic system (4) is progressively stable if the modulus of all eigenvalues of matrix A is not more than 1;*
*(2)*  *In case there is a matrix (Q > 0), so that the Lyapunov equation (A^T^PA − P = −Q) has a unique positive solution (P), system (4) is asymptotically stable.*

**Proof** **of** **Lemma** **1**(**1**)**.**  Set $V\left(x\left(k\right)\right)=x^{T}\left(k\right)x\left(k\right)$, and then the tiny variables of V(x(k)) can be calculated as follows:

(5) $$\begin{array}{l} \Delta V\left(x\left(k\right)\right)=V\left(x\left(k+1\right)\right)-V\left(x\left(k\right)\right)\\ \;\;\;\;\;=x^{T}\left(k+1\right)x\left(k+1\right)-x^{T}\left(k\right)x\left(k\right)\\ \;\;\;\;\;=x^{T}\left(k\right)A^{T}Ax\left(k\right)-x^{T}\left(k\right)x\left(k\right)\\ \;\;\;\;\;\leq \left(\lambda_{\max}\left(A^{T}A\right)-1\right)x^{T}\left(k\right)x\left(k\right) \end{array}$$

Because all the eigenvalues of matrix *A* have a value of modulo less than 1, all the eigenvalues of matrix $A^{T}A$ are integers, which are less than 1; therefore, ΔV(x(k))<0, and system (4) is asymptotically stable. □

**Proof** **of** **Lemma** **1**(**2**)**.**  Set $V\left(x\left(k\right)\right)=x^{T}\left(k\right)Px\left(k\right)$, where P is a positive definite matrix, given that $A^{T}PA-P=-Q$, and then the tiny variables of V(x(k)) can be calculated as follows:

(6) $$\begin{array}{l} \Delta V\left(x\left(k\right)\right)=V\left(x\left(k+1\right)\right)-V\left(x\left(k\right)\right)\\ \;\;\;\;\;\;=x^{T}\left(k+1\right)Px\left(k+1\right)-x^{T}\left(k\right)Px\left(k\right)\\ \;\;\;\;\;\;=x^{T}\left(k\right)A^{T}PAx\left(k\right)-x^{T}\left(k\right)Px\left(k\right)\\ \;\;\;\;\;\;=x^{T}\left(k\right)\left(A^{T}PA-P\right)x\left(k\right)\\ \;\;\;\;\;\;=-x^{T}\left(k\right)Qx\left(k\right)<0 \end{array}$$

Furthermore, $\underset{x\to \infty }{\lim}x\left(k\right)=0$; hence, system (4) is asymptotically stable. □

Based on the proof processes for the stability of linear discrete systems as related in **Lemma 1**, the determination processes for the stability of nonlinear discrete systems can be given through **Lemma 2**, which is described as follows:

**Lemma** **2.** *For a nonlinear discrete system* (x(k+1)=f(x(k)),k=0,1,2⋯)*, let* $x_{e}=0$ *(i.e.,* f(0)=0*) be the equilibrium point of the proposed system. Provided that the scalar function* x(k)=0 *concerning* V(x(k)) *satisfies the following:*

*(1)*  V(x(k))>0*,*
*(2)*  ΔV(x(k))=V(x(k+1))−V(x(k))<0*.*

*then* $x_{e}=0$ *is progressively stable.*

**Proof** **of** **Lemma** **2.** For condition (1), let $V\left(x\left(k\right)\right)=x^{T}\left(k\right)x\left(k\right)$, and in the case of x≠0, V(x(k))>0, the first condition is proven. For condition (2), let $V\left(x\left(k\right)\right)=x^{T}\left(k\right)Px\left(k\right)$. We can prove that P is a positive definite matrix from **Lemma 1.** Moreover, $A^{T}PA-P=-Q$, and Q>0; subsequently, the small changes (V(x(k))) can be described (ΔV(x(k))), which are calculated as follows:

(7) $$\begin{array}{l} \Delta V\left(x\left(k\right)\right)=V\left(x\left(k+1\right)\right)-V\left(x\left(k\right)\right)\\ \;\;\;\;\;\;=x^{T}\left(k+1\right)Px\left(k+1\right)-x^{T}\left(k\right)Px\left(k\right)\\ \;\;\;\;\;\;=x^{T}\left(k\right)A^{T}PAx\left(k\right)-x^{T}\left(k\right)Px\left(k\right)\\ \;\;\;\;\;\;=x^{T}\left(k\right)\left(A^{T}PA-P\right)x\left(k\right)\\ \;\;\;\;\;\;=-x^{T}\left(k\right)Qx\left(k\right)<0 \end{array}$$

□

Thus, the proof of condition (2) is complete. Based on the above, it is concluded that nonlinear system (4) is asymptotically stable at the origin point.

Thus, having proved **Lemma 1** and **Lemma 2**, the proof of Theorem 1 can be obtained, which is as follows:

**Proof** **of** **Theorem** **1.** According to Equations (1) and (2), Equation (3) can be calculated as follows:

(8) $$e\left(k+1\right)=H\left(X_{m}\left(k+1\right)\right)-Y\left(k+1\right)=H\left(F\left(X\left(k\right)\right)\right)-Q\left(Y\left(k\right)\right)-G\left(X\left(k\right),Y\left(k\right)\right)$$

where Q(Y(k))=F(Y(k))+U(k), and U(k) is a control function, which be represented as follows:

(9) U(k)=H(F(X(k)))−F(Y(k))+(1−η)G(X(k),Y(k))

Then, Equation (9) can be simplified as the following equation:

(10) e(k+1)=ηG(X(k),Y(k))=ηe(k)

Denote the scaled function of the nonlinear error system (e(k)=0) represented as $V\left(e\left(k\right)\right)=e{\left(k\right)}^{T}e\left(k\right)$, and then the ΔV(e(k)) is calculated as follows:

(11) $$\begin{array}{l} \Delta V\left(e\left(k\right)\right)=V\left(e\left(k+1\right)\right)-V\left(e\left(k\right)\right)\\ \;\;\;\;=e{\left(k+1\right)}^{T}e\left(k+1\right)-e{\left(k\right)}^{T}e\left(k\right)\\ \;\;\;\;=\eta e{\left(k\right)}^{T}\eta e\left(k\right)-e{\left(k\right)}^{T}e\left(k\right)\\ \;\;\;\;=\eta^{2}e{\left(k\right)}^{T}e\left(k\right)-e{\left(k\right)}^{T}e\left(k\right)\\ \;\;\;\;=\left(\eta^{2}-1\right)e{\left(k\right)}^{T}e\left(k\right) \end{array}$$

Therefore, when the parameter η satisfies the condition $\eta^{2}<1$, ΔV(e(k))<0; hence, V(e(k+1))/V(e(k))<1 and $\underset{k\to \infty }{\lim}V\left(e\left(k\right)\right)=\underset{k\to \infty }{\lim}e^{T}\left(k\right)e\left(k\right)=0$. □

Furthermore, $\underset{k\to \infty }{\lim}e\left(k\right)=0$. Thus, according to **Lemma 2**, the nonlinear error system (e(k)) is asymptotically stable when e=0, and, in turn, the drive and response systems are asymptotically synchronized.

## 3. Analysis of the Dynamical Behavior of 3D Discrete Chaotic Systems and Implementation of The Proposed Generalized Synchronization Method by Incorporating Parameter Control

### 3.1. The Proposed New 3D Discrete Chaotic System

**Figure 1:**
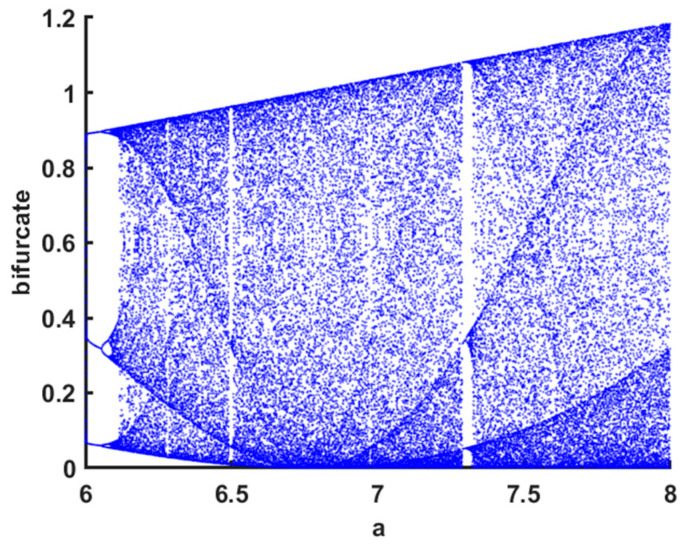
Bifurcation diagram with the *a* of the proposed system (12).

A new 3D discrete chaotic system (12) is proposed in this paper, which is described as follows:

(12) $$\left\{ \begin{array}{l} x_{1}\left(k+1\right)=0.665x_{1}^{2}\left(k\right)+3.5x_{1}\left(k\right)-0.5\\ x_{2}\left(k+1\right)=0.82x_{2}^{2}\left(k\right)-2.34\\ x_{3}\left(k+1\right)=ax_{3}\left(k\right){\left(1-x_{3}\left(k\right)\right)}^{2} \end{array}\right.$$

where the $x_{1}$, $x_{2}$, and $x_{3}$ are iterative variables, and *a* is a parameter variable. The bifurcation diagram with the *a* of system (12) is represented by Figure 1. It is clear from the bifurcation diagram that the system is chaotic when *a* = 6.53, and after 1000 iterations, the Lyapunov exponents of system (12) are 0.7296, 0.1650, and 0.6226, which are all positive; thus, system (12) is a hyperchaotic system.

**Figure 2:**
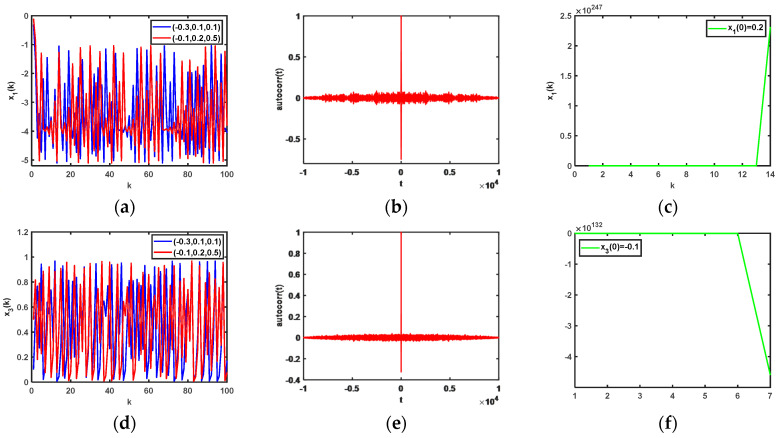
Output of chaotic sequences with different initial values of state variables and their autocorrelations: (**a**) output of $x_{1}\left(k\right)$ when the initial values of the system are different; (**b**) autocorrelations of $x_{1}\left(k\right)$ when $x_{1}\left(0\right)=-0.3$, $x_{2}\left(0\right)=0.1$, and $x_{3}\left(0\right)=0.1$; (**c**) output chaotic sequences of $x_{1}\left(k\right)$ when $x_{1}\left(0\right)=0.2$; (**d**) output of $x_{3}\left(k\right)$ when the initial values of the system are different; (**e**) autocorrelations of the output chaotic sequences ($x_{3}\left(k\right)$) when $x_{1}\left(0\right)=-0.1$, $x_{2}\left(0\right)=0.2$, and $x_{3}\left(0\right)=0.5$; (**f**) output chaotic sequences of $x_{3}\left(k\right)$ when $x_{3}\left(0\right)=0.1$.

**Figure 3:**
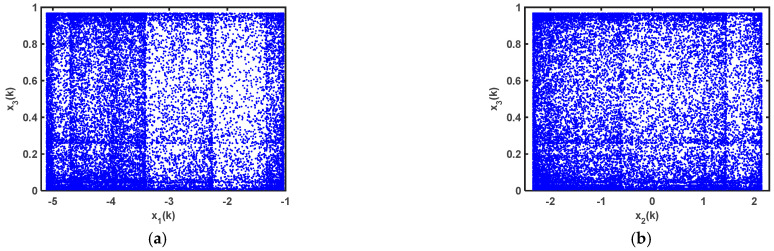

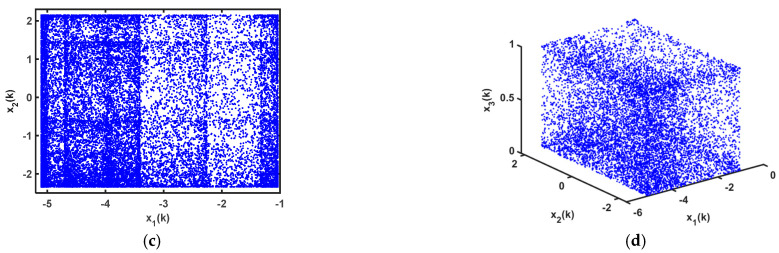
Phase diagrams of proposed 3D hyperchaotic mapping (5): (**a**) $x_{1}-x_{3}$; (**b**) $x_{2}-x_{3}$; (**c**) $x_{1}-x_{2}$; (**d**) $x_{1}-x_{2}-x_{3}$.

Based on 64-bit Matlab software and double-floating-point representation, the initial values of the state variables of the chaotic system are set differently. The output chaotic sequences and their autocorrelation are shown in Figure 2, from which we can clearly see in Figure 2a,b,d,e that the chaotic sequences $x_{1}\left(k\right)$ and $x_{3}\left(k\right)$ have no periodicity, and Figure 2a,d demonstrate the proposed chaotic sequences with an initial value sensitivity. The initial values of the sequences are set as $x_{1}(0)=0.2$ and $x_{3}\left(0\right)=-0.1$, respectively, and the corresponding chaotic iteration diagrams are shown in Figure 2c,f, respectively, which show that system (12) is not in a chaotic state in this case. Therefore, the initial values of the chaotic system are what affect the output states of system (12). In addition, the chaotic attractor phase diagrams of the proposed 3D hyperchaotic mapping are shown in Figure 3 as $x_{1}(0)=-0.3$, $x_{2}\left(0\right)=0.1$, and $x_{3}\left(0\right)=0.1$.

### 3.2. Implementation of Proposed Generalized Synchronization Method Incorporating Parameter Control

Let us assume that the system of responding systems of the drive system (12) is as follows:

(13) $$\left\{ \begin{array}{l} y_{1}\left(k+1\right)=0.665y_{1}^{2}\left(k\right)+3.5y_{1}\left(k\right)-0.5+u_{1}\left(k\right)\\ y_{2}\left(k+1\right)=0.82y_{2}^{2}\left(k\right)-2.34+u_{2}\left(k\right)\\ y_{3}\left(k+1\right)=6.53y_{3}\left(k\right){\left(1-y_{3}\left(k\right)\right)}^{2}+u_{3}\left(k\right) \end{array}\right.$$

where $y_{1}$, $y_{2}$, and $y_{3}$ are iteration variables, and based on **Theorem 1**, the system errors are calculated as follows:

(14) $$\left\{ \begin{array}{l} e_{1}\left(k\right)=h_{1}x_{1}-y_{1}\\ e_{2}\left(k\right)=h_{2}x_{2}-y_{2}\\ e_{3}\left(k\right)=h_{3}x_{3}-y_{3} \end{array}\right.$$

Then, the error system equation can be expressed as follows:

(15) $$\left\{ \begin{array}{l} e_{1}\left(k+1\right)=h_{1}\left(0.665x_{1}^{2}\left(k\right)+3.5x_{1}\left(k\right)-0.5\right)-\left(0.665y_{1}^{2}\left(k\right)+3.5y_{1}\left(k\right)-0.5+u_{1}\left(k\right)\right)\\ e_{2}\left(k+1\right)=h_{2}\left(0.82x_{2}^{2}\left(k\right)-2.34\right)-\left(0.82y_{2}^{2}\left(k\right)-2.34+u_{2}\left(k\right)\right)\\ e_{3}\left(k+1\right)=h_{3}\left(6.53x_{3}\left(k\right){\left(1-x_{3}\left(k\right)\right)}^{2}\right)-\left(6.53y_{3}\left(k\right){\left(1-y_{3}\left(k\right)\right)}^{2}+u_{3}\left(k\right)\right) \end{array}\right.$$

Let the control function be represented by Equation (16):

(16) $$\left\{ \begin{array}{l} u_{1}(k)=h_{1}\left(0.665x_{1}^{2}\left(k\right)+3.5x_{1}\left(k\right)-0.5\right)-\left(0.665y_{1}^{2}\left(k\right)+3.5y_{1}\left(k\right)-0.5\right)-\eta \left(h_{1}x_{1}\left(k\right)-y_{1}\left(k\right)\right)\\ u_{2}(k)=h_{2}\left(0.82x_{2}^{2}\left(k\right)-2.34\right)-\left(0.82y_{2}^{2}\left(k\right)-2.34\right)-\eta \left(h_{2}x_{2}\left(k\right)-y_{2}\left(k\right)\right)\\ u_{3}(k)=h_{3}\left(6.53x_{3}\left(k\right){\left(1-x_{3}\left(k\right)\right)}^{2}\right)-\left(6.53y_{3}\left(k\right){\left(1-y_{3}\left(k\right)\right)}^{2}\right)-\eta \left(h_{3}x_{3}\left(k\right)-y_{3}\left(k\right)\right) \end{array}\right.$$

Thus, system (14) can be simplified to expression (17):

(17) $$\left\{ \begin{array}{l} e_{1}\left(k+1\right)=\eta e_{1}\left(k\right)\\ e_{2}\left(k+1\right)=\eta e_{2}\left(k\right)\\ e_{3}\left(k+1\right)=\eta e_{3}\left(k\right) \end{array}\right.$$

Let the Lyapunov exponent function of system (17) be represented by the following expressions:

(18) $$V(e(k))=\frac{1}{2}\left(e_{1}^{2}(k)+e_{2}^{2}(k)+e_{3}^{2}(k)\right)\geq 0$$

(19) $$\begin{array}{l} \Delta V(e(k))=V(e(k+1))-V(e(k))\\ \;\;\;\;=\frac{1}{2}\left(e_{1}^{2}(k+1)+e_{2}^{2}(k+1)+e_{3}^{2}(k+1)\right)-\frac{1}{2}\left(e_{1}^{2}(k)+e_{2}^{2}(k)+e_{3}^{2}(k)\right)\\ \;\;\;\;=\frac{\eta^{2}-1}{2}\left(e_{1}^{2}(k)+e_{2}^{2}(k)+e_{3}^{2}(k)\right) \end{array}$$

It is obvious that when the parameter is |η|<1, then ΔV(e(k))<0, and the error system is progressively stable at e=0, according to **Lemma 1**. The zero solution of error system (13) is asymptotically stable so that generalized chaotic synchronization can be achieved. In summary, the incorporation of the error-system-feedback coefficient (η) into the design of the controller for generalized synchronization in this paper makes the design of the controller more flexible.

Let the invertible transformation equation be H(x(k))=Ax(k), and matrix *A* is as follows:

(20) $$A=\left[\begin{array}{ccc} 2 & 1 & 1\\ 1 & 2 & 1\\ 1 & 1 & 2 \end{array}\right]$$

**Figure 4:**
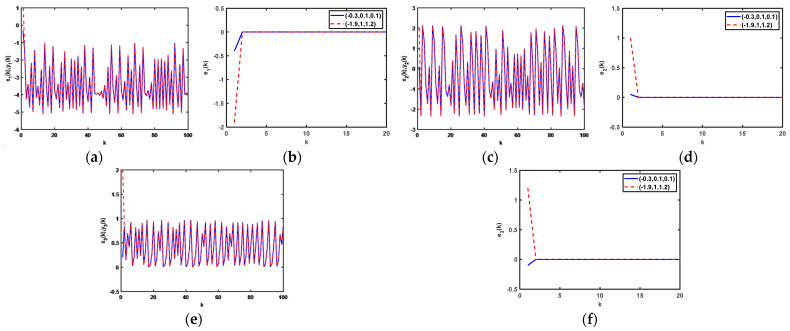
(**a**) Dynamical curves of status variables $x_{1}\left(k\right)$ and $y_{1}\left(k\right)$; (**b**) dynamical curves of $e_{1}\left(k\right)$; (**c**) dynamical curves of status variables $x_{2}\left(k\right)$ and $y_{2}\left(k\right)$; (**d**) dynamical curves of $e_{2}\left(k\right)$; (**e**) dynamical curves of status variables $x_{3}\left(k\right)$ and $y_{3}\left(k\right)$; (**f**) dynamical curves of $e_{3}\left(k\right)$.

In the initial condition for the iterative variables $x_{1}\left(0\right)=-0.3$, $x_{2}\left(0\right)=0.1$, and $x_{3}\left(0\right)=0.1$, with 1000 iterations, the dynamical curves of the status variables $x_{1}\left(k\right)$, $y_{1}\left(k\right)$, $x_{2}\left(k\right)$, $y_{2}\left(k\right)$, $x_{3}\left(k\right)$, and $y_{3}\left(k\right)$ are displayed in Figure 4a,c,e, respectively, whereas the dynamical curves of $e_{1}\left(k\right)$, $e_{2}\left(k\right)$, and $e_{3}\left(k\right)$ are represented in Figure 4b,d,f, respectively, from which it can be seen that the difference in the initial values does not affect the synchronization time.

## 4. Implementation of a Universal Synchronization Method with Parameter Control and Analysis of The Dynamic Behavior of the Proposed 6D Discrete Chaotic System

### 4.1. The Proposed 6D Discrete Chaotic System

High-dimensional chaotic systems have more complex dynamics than low-dimensional chaotic systems; thus, they are better able to resist the degradation of dynamics caused by the limited accuracy of computers. In this paper, a new 6D discrete chaotic system (21) is constructed by expanding on system (12), which can be presented as follows:

(21) $$\left\{ \begin{array}{l} x_{1}(k+1)=0.665x_{1}{\left(k\right)}^{2}+ax_{1}(k)-0.5;\\ x_{2}(k+1)=0.82x_{2}{\left(k\right)}^{2}-b;\\ x_{3}(k+1)=cx_{3}(k){\left(1-x_{3}(k)\right)}^{2};\\ x_{4}(k+1)=-3x_{4}^{2}(k)+dx_{4}(k)+0.18;\\ x_{5}(k+1)=4x_{5}(k)(1-x_{5}(k));\\ x_{6}(k+1)=4x_{6}(k)(1-0.5x_{6}(k)); \end{array}\right.$$

where *a* = 3.5, *b* = −2.34, *c* = 6.53, and *d* = 3.46, after 1000 iterations, and the Lyapunov exponents of the proposed system are 1.0227, 0.3837, −0.2378, −0.2999, 0.4955, and 1.7245, respectively; thus, system (21) is a hyperchaotic system because the four Lyapunov exponents are positive.

**Figure 5:**
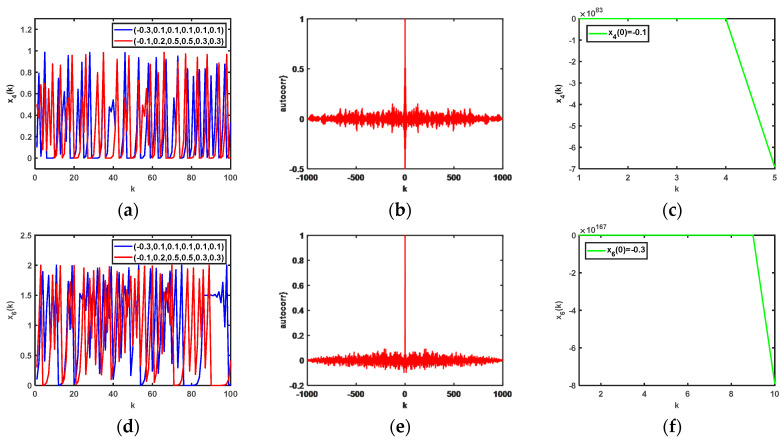
Output of chaotic sequences with different initial values of state variables and their autocorrelations: (**a**) output of $x_{4}\left(k\right)$ when the initial values of the system are different; (**b**) autocorrelations of $x_{4}\left(k\right)$ when $x_{1}\left(0\right)=-0.3$, $x_{2}\left(0\right)=0.1$, $x_{3}\left(0\right)=0.1$, $x_{4}\left(0\right)=0.1$, $x_{5}\left(0\right)=0.1$, and $x_{6}\left(0\right)=0.1$; (**c**) output chaotic sequences of $x_{4}\left(k\right)$ when $x_{4}\left(0\right)=-0.1$; (**d**) output of $x_{6}\left(k\right)$ when the initial values of the system are different; (**e**) autocorrelations of the output chaotic sequences of $x_{6}\left(k\right)$ when $x_{1}\left(0\right)=-0.1$, $x_{2}\left(0\right)=0.2$, $x_{3}\left(0\right)=0.5$, $x_{4}\left(0\right)=0.5$, $x_{5}\left(0\right)=0.3$, and $x_{6}\left(0\right)=0.3$; (**f**) output chaotic sequences of $x_{6}\left(k\right)$ when $x_{6}\left(0\right)=-0.3$.

**Figure 6:**
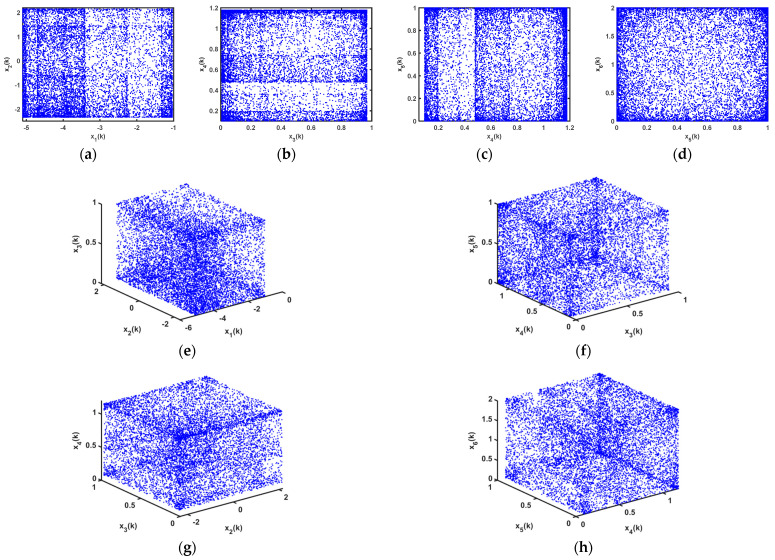
Phase diagrams of proposed 6D hyperchaotic system (14): (**a**) $x_{1}\left(k\right)-x_{2}\left(k\right)$; (**b**) $x_{3}\left(k\right)-x_{4}\left(k\right)$; (**c**) $x_{4}\left(k\right)-x_{5}\left(k\right)$; (**d**) $x_{5}\left(k\right)-x_{6}\left(k\right)$; (**e**) $x_{1}\left(k\right)-x_{2}\left(k\right)-x_{3}\left(k\right)$; (**f**) $x_{3}\left(k\right)-x_{4}\left(k\right)-x_{5}\left(k\right)$; (**g**) $x_{2}\left(k\right)-x_{3}\left(k\right)-x_{4}\left(k\right)$; (**h**) $x_{4}\left(k\right)-x_{5}\left(k\right)-x_{6}\left(k\right)$.

Based on 64-bit Matlab software and double-floating-point representation, for different initial values of the state variables, the output of chaotic sequences and their autocorrelations are shown in Figure 5, in which Figure 5a,b,d,e prove that the chaotic sequences $x_{4}\left(k\right)$ and $x_{6}\left(k\right)$ have no periodicity, and Figure 5a,d demonstrate the proposed chaotic sequences with an initial value sensitivity. The initial values of the sequences were set at $x_{4}\left(0\right)=-0.1$, and $x_{6}\left(0\right)=-0.3$, respectively, and the corresponding chaotic iteration diagrams are shown in Figure 5c,f, respectively, which show that system (21) is not in a chaotic state in this case. Therefore, the initial values of the chaotic system are what affect the output states of system (21). In addition, the chaotic attractor phase diagrams of the proposed 6D hyperchaotic mapping are shown in Figure 6 as $x_{1}\left(0\right)=-0.3$, $x_{2}\left(0\right)=0.1$, $x_{3}\left(0\right)=0.1$, $x_{4}\left(0\right)=0.1$, $x_{5}\left(0\right)=0.1$, and $x_{6}\left(0\right)=0.1$.

### 4.2. Implementation of Generalised Synchronisation Incorporating Parameter Control

Let the corresponding system of driving system (21) be considered as follows:

(22) $$\left\{ \begin{array}{l} y_{1}\left(k+1\right)=0.665y_{1}^{2}\left(k\right)+3.5y_{1}\left(k\right)-0.5+u_{1}\left(k\right);\\ y_{2}\left(k+1\right)=0.82y_{2}^{2}\left(k\right)-2.34+u_{2}\left(k\right)\\ y_{3}\left(k+1\right)=6.53y_{3}\left(k\right){\left(1-y_{3}\left(k\right)\right)}^{2}+u_{3}\left(k\right)\\ y_{4}(k+1)=-3y_{4}^{2}(k)+3.46y_{4}(k)+0.18+u_{4}\left(k\right)\\ y_{5}(k+1)=4y_{5}(k)(1-y_{5}(k))+u_{5}\left(k\right)\\ y_{6}(k+1)=4y_{6}(k)(1-0.5y_{6}(k))+u_{6}\left(k\right) \end{array}\right.$$

Equation (23) is obtained from **Lemma 2**:

(23) $$\left\{ \begin{array}{l} e_{1}\left(k\right)=h_{1}x_{1}-y_{1}\\ e_{2}\left(k\right)=h_{2}x_{2}-y_{2}\\ e_{3}\left(k\right)=h_{3}x_{3}-y_{3}\\ e_{4}\left(k\right)=h_{4}x_{4}-y_{4}\\ e_{5}\left(k\right)=h_{5}x_{5}-y_{5}\\ e_{6}\left(k\right)=h_{6}x_{6}-y_{6} \end{array}\right.$$

The equation of the error system can then be expressed as follows:

(24) $$\left\{ \begin{array}{l} e_{1}\left(k+1\right)=h_{1}\left(0.665x_{1}^{2}\left(k\right)+3.5x_{1}\left(k\right)-0.5\right)-\left(0.665y_{1}^{2}\left(k\right)+3.5y_{1}\left(k\right)-0.5+u_{1}\left(k\right)\right);\\ e_{2}\left(k+1\right)=h_{2}\left(0.82x_{2}^{2}\left(k\right)-2.34\right)-\left(0.82y_{2}^{2}\left(k\right)-2.34+u_{2}\left(k\right)\right)\\ e_{3}\left(k+1\right)=h_{3}\left(6.53x_{3}\left(k\right){\left(1-x_{3}\left(k\right)\right)}^{2}\right)-\left(6.53y_{3}\left(k\right){\left(1-y_{3}\left(k\right)\right)}^{2}+u_{3}\left(k\right)\right)\\ e_{4}\left(k+1\right)=h_{4}\left(-3x_{4}^{2}(k)+3.46x_{4}(k)+0.18\right)-\left(-3y_{4}^{2}(k)+3.46y_{4}(k)+0.18+u_{4}\left(k\right)\right)\\ e_{5}\left(k+1\right)=h_{5}\left(4x_{5}(k)(1-x_{5}(k))\right)-\left(4y_{5}(k)(1-y_{5}(k))+u_{5}\left(k\right)\right)\\ e_{6}\left(k+1\right)=h_{6}\left(4x_{6}(k)(1-0.5x_{6}(k))\right)-\left(4y_{6}(k)(1-0.5y_{6}(k))+u_{6}\left(k\right)\right) \end{array}\right.$$

Let the control functions be calculated as follows:

(25) $$\left\{ \begin{array}{l} u_{1}\left(k\right)=h_{1}\left(0.665x_{1}^{2}\left(k\right)+3.5x_{1}\left(k\right)-0.5\right)-\left(0.665y_{1}^{2}\left(k\right)+3.5y_{1}\left(k\right)-0.5\right)+\eta \left(h_{1}x_{1}\left(k\right)-y_{1}\left(k\right)\right)\\ u_{2}\left(k\right)=h_{2}\left(0.82x_{2}^{2}\left(k\right)-2.34\right)-\left(0.82y_{2}^{2}\left(k\right)-2.34\right)+\eta \left(h_{2}x_{2}\left(k\right)-y_{2}\left(k\right)\right)\\ u_{3}\left(k\right)=h_{3}\left(6.53x_{3}\left(k\right){\left(1-x_{3}\left(k\right)\right)}^{2}\right)-\left(6.53y_{3}\left(k\right){\left(1-y_{3}\left(k\right)\right)}^{2}\right)+\eta \left(h_{3}x_{3}\left(k\right)-y_{3}\left(k\right)\right)\\ u_{4}\left(k\right)=h_{4}\left(-3x_{4}^{2}(k)+3.46x_{4}(k)+0.18\right)-\left(-3y_{4}^{2}(k)+3.46y_{4}(k)+0.18+u_{4}\left(k\right)\right)+\eta \left(h_{4}x_{4}\left(k\right)-y_{4}\left(k\right)\right)\\ u_{5}\left(k\right)=h_{5}\left(4x_{5}(k)(1-x_{5}(k))\right)-\left(4y_{5}(k)(1-y_{5}(k))+u_{5}\left(k\right)\right)+\eta \left(h_{5}x_{5}\left(k\right)-y_{5}\left(k\right)\right)\\ u_{6}\left(k\right)=h_{6}\left(4x_{6}(k)(1-0.5x_{6}(k))\right)-\left(4y_{6}(k)(1-0.5y_{6}(k))+u_{6}\left(k\right)\right)+\eta \left(h_{6}x_{6}\left(k\right)-y_{6}\left(k\right)\right) \end{array}\right.$$

Then, system (24) can be expressed as follows:

(26) $$\left\{ \begin{array}{l} e_{1}\left(k+1\right)=\eta e_{1}\left(k\right)\\ e_{2}\left(k+1\right)=\eta e_{2}\left(k\right)\\ e_{3}\left(k+1\right)=\eta e_{3}\left(k\right)\\ e_{4}\left(k+1\right)=\eta e_{4}\left(k\right)\\ e_{5}\left(k+1\right)=\eta e_{5}\left(k\right)\\ e_{6}\left(k+1\right)=\eta e_{6}\left(k\right) \end{array}\right.$$

Consider the Lyapunov exponent functions of system (25) as follows:

(27) $$V(e(k))=\frac{1}{2}\left(e_{1}^{2}(k)+e_{2}^{2}(k)+e_{3}^{2}(k)+e_{4}^{2}(k)+e_{5}^{2}(k)+e_{6}^{2}(k)\right)\geq 0$$

(28) $$\Delta V(e(k))=\frac{\eta^{2}-1}{2}\left(e_{1}^{2}(k)+e_{2}^{2}(k)+e_{3}^{2}(k)+e_{4}^{2}(k)+e_{5}^{2}(k)+e_{6}^{2}(k)\right)\leq 0$$

Obviously, according to **Lemma 2**, when the parameter |η|<1 and, hence, the error system is progressively stable at e=0, the zero solution of the error system (25) is progressively stable, and as a result, the generalized chaotic synchronization can be implemented.

Let the invertible transformation function be H(x(k))=Ax(k), and let the coefficient matrix *A* be expressed as follows:

(29) $$A=\left[\begin{array}{cccccc} 2 & 1 & 1 & 1 & 1 & 1\\ 1 & 2 & 1 & 1 & 1 & 1\\ 1 & 1 & 2 & 1 & 1 & 1\\ 1 & 1 & 1 & 2 & 1 & 1\\ 1 & 1 & 1 & 1 & 2 & 1\\ 1 & 1 & 1 & 1 & 1 & 2 \end{array}\right]$$

**Figure 7:**
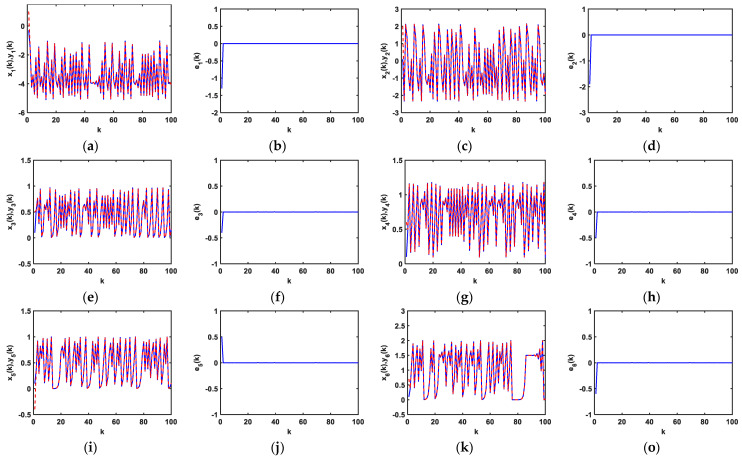
Dynamical diagrams of status variables with *k*: System (21): (**a**) $\left(x_{1}\left(k\right),y_{1}\left(k\right)\right)$; (**b**) $e_{1}\left(k\right)$; (**c**) $\left(x_{2}\left(k\right),y_{2}\left(k\right)\right)$; (**d**) $e_{2}\left(k\right)$; System (22): (**e**) $\left(x_{3}\left(k\right),y_{3}\left(k\right)\right)$; (**f**) $e_{3}\left(k\right)$; (**g**) $\left(x_{4}\left(k\right),y_{4}\left(k\right)\right)$; (**h**) $e_{4}\left(k\right)$; System (23): (**i**) $\left(x_{5}\left(k\right),y_{5}\left(k\right)\right)$; (**j**) $e_{5}\left(k\right)$; (**k**) $\left(x_{6}\left(k\right),y_{6}\left(k\right)\right)$; (**l**) $e_{6}\left(k\right)$.

Moreover, set the initial conditions of the proposed driving system (21) as $x_{1}(0)=-0.3$, $x_{2}\left(0\right)=0.1$, $x_{3}\left(0\right)=0.2$, $x_{4}\left(0\right)=0.01$, $x_{5}\left(0\right)=0.1$, and $x_{6}\left(0\right)=0.1$. After 1000 iterations, the dynamical diagrams of the change in the status of the iterations as *k* changes for systems (21), (22), and (24) are shown in Figure 7. The Figure 7a–d describe the dynamical diagrams of system (21), and the dynamical diagrams of system (22) are shown in Figure 7e–h, and Figure 7i–l display the dynamical diagrams of system (23).

## 5. Cryptographic Transmission System for Digital Images Based on Proposed Generalized Chaos Synchronization Approach

### 5.1. Cryptographic Transmission System for Digital Images

**Figure 8:**
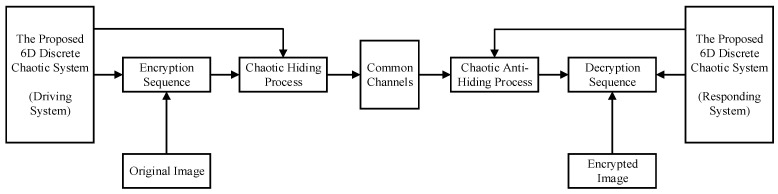
Framework diagram of proposed encrypted transmission system.

The framework diagram of the proposed encryption and decryption transmission system constructed in this paper is shown in Figure 8.

The proposed system is designed for the encrypted transmission of digital images with pixel matrix values of $R_{m\times n}\left(x,y\right)$, $G_{m\times n}\left(x,y\right)$, and $B_{m\times n}\left(x,y\right)$ for each component of the color digital image, where the number of pixels in the image is n×m. Then, matrix components are converted into a sequence of integer values in row order, and the pixel values are selected in the range (0,255), converting each pixel value into an 8-bit binary number. Based on the above operations, the binary sequences R(j), G(j), and B(j), which are based on the color image, can be obtained, where j∈(0,m×n×8).

The output sequences (x(k)) of the proposed 6D generalized discrete chaotic system are quantized by the region to generate binary sequences for encryption. Therefore, the quantification process can be represented by the following equation:

(30) $$P\left(j\right)=P_{0-1}\left[x\left(i\right)\right]=\left\{ \begin{array}{l} 1,\;x\left(i\right)\in \overset{2^{m}-1}{\underset{n=0}{\cup }}I_{2n}^{m}\\ 0,\;x\left(i\right)\in \overset{2^{m}-1}{\underset{n=0}{\cup }}I_{2n+1}^{m} \end{array}\right.;n=0,1,2\cdots$$

where m is an arbitrary integer greater than 0, and $I_{0}^{m},I_{1}^{m},I_{2}^{m}\cdots$ are denoted as $2^{m}$ consecutive equal intervals on the interval of a range of real-valued sequences. If the output value of a chaotic sequence is in the odd interval, then it outputs 0, and if it is in the even interval, then it outputs 1.

The workflow of the whole system is as follows, and the process of encryption on the transmitter side of the proposed system consists of the following parts:

**Step 1:** The chaotic sequences generated by the proposed 6D discrete chaotic system are quantized and denoted as $P_{1}\left(j\right)$, $P_{2}\left(j\right)$, $P_{3}\left(j\right)$, and $P_{4}\left(j\right)$. Furthermore, the three color components of the original image are encrypted with chaotic sequences, and the calculation formula is Equation (31):

(31) $$\left\{ \begin{array}{l} E_{R}\left(j\right)=R\left(j\right)⊕P_{1}\left(j\right)\\ E_{G}\left(j\right)=G\left(j\right)⊕P_{2}\left(j\right)\\ E_{B}\left(j\right)=B\left(j\right)⊕P_{3}\left(j\right) \end{array}\right.$$

**Step 2:** The encrypted sequences of the three color components are combined into E(k) using Equation (32):

(32) $$\begin{array}{l} E_{R}\left(j\right)\\ E_{G}\left(j\right)\\ E_{B}\left(j\right) \end{array}\}\Rightarrow E\left(k\right),\;k\in \left(0,3\times j\right)$$

**Step 3:** Chaotic hiding of E(k) with the chaotic sequence $P_{4}\left(j\right)$. The resulting mixed signal (S(k)) is transmitted in the common channel and is calculated as the following equation:

(33) S(k)=E(k)+P(k)

On the receiving side of the proposed system, the response system will be in general synchronization with the driver system. Furthermore, the receiver will be able to decode all the state variables of the sender. Similarly, there are several parts to the decryption processes for the receiver of the proposed system.

**Step 1:** Reconstructing the chaotic signal (y(k)), the sequences generated after quantization are denoted as $P_{1}{\left(j\right)}^{\prime }$, $P_{2}{\left(j\right)}^{’}$, $P_{3}{\left(j\right)}^{\prime }$, and $P_{4}{\left(3\times j\right)}^{\prime }$, as we can see from Figure 8. The decryption process is the inverse of the encryption process; thus, it is important to perform the anti-hiding operation on the signal S(k) to obtain $E^{\prime }\left(k\right)$, which is calculated as follows:

(34) $$S\left(k\right)-P{\left(k\right)}_{4}=E^{\prime }\left(k\right)$$

**Step 2:** Decompose $E^{\prime }\left(k\right)$ into three color components. The formulation is calculated as Equation (35), and therefore the encrypted image is decoded using Equation (36):

(35) $$E^{\prime }\left(k\right)\Rightarrow \left\{ \begin{array}{l} E_{R}^{\prime }\left(j\right)\\ E_{G}^{\prime }\left(j\right)\\ E_{B}^{\prime }\left(j\right) \end{array}\right.$$

(36) $$\left\{ \begin{array}{l} E_{R}^{\prime }\left(j\right)⊕P_{1}^{\prime }{\left(j\right)}_{}=R^{\prime }\left(j\right)\\ E_{G}^{\prime }\left(j\right)⊕P_{2}^{\prime }\left(j\right)=G^{\prime }\left(j\right)\\ E_{B}^{\prime }\left(j\right)⊕P_{3}^{\prime }\left(j\right)=B^{\prime }\left(j\right) \end{array}\right.$$

**Figure 9:**
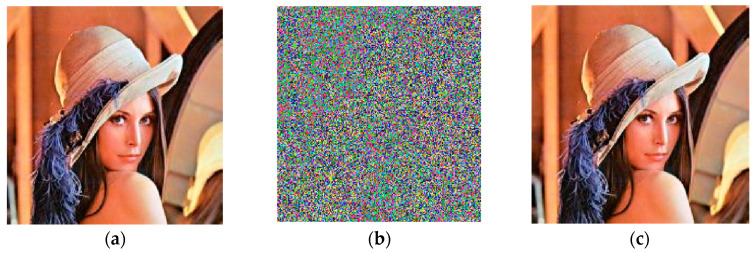
Results of proposed encryption and decryption transmission system: (**a**) original Lena; (**b**) encrypted Lena; (**c**) decrypted Lena.

The standard Lena (256 × 256) image was used as an example for the encryption and decryption processes, and the results of the operation are shown in Figure 9.

### 5.2. Time Complexity

As can be seen from the encryption process in Section 5.1, the entire encryption algorithm consists of simple operations, such as addition, subtraction, and iso-or. However, in the process of encrypting a three-dimensional matrix of the size 8 × *m* × *n*, it takes about $n^{2}$ operations to complete the bit-level operation of the encryption of the xor operation. Hence, the time complexity of the proposed algorithm in this paper is $T\left(n\right)=O\left(n^{2}\right)$.

## 6. Security Analyses of Proposed Scheme

Security analyses of the transmission system for the proposed digital image encryption and decryption based on generalized chaotic synchronization are performed in this paper, which consist of image encryption histogram analysis, key space analysis, key sensitivity analysis, and correlation analysis.

### 6.1. Histogram Analysis of Encrypted Image

**Figure 10:**
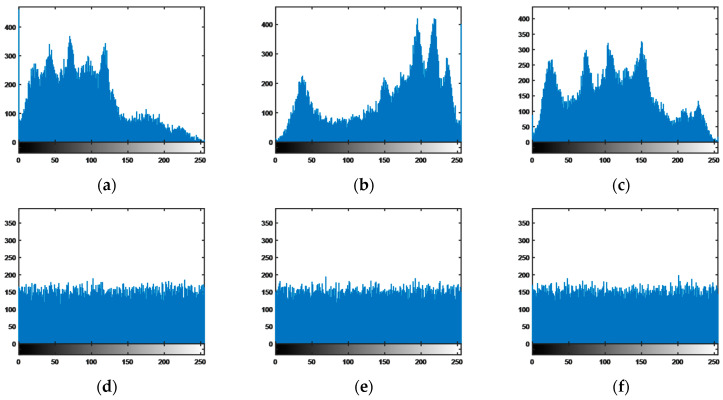
Histograms of different components: Original Lena: (**a**) R component of original Lena; (**b**) G component of original Lena; (**c**) B component of original Lena; Encrypted Lena: (**d**) R component of encrypted Lena; (**e**) G component of encrypted Lena; (**f**) B component of encrypted Lena.

A color histogram is a presentation of the statistical characteristics and distribution of the image pixels and is analyzed in terms of three colors: R, G, and B. As can be seen from the simulation results in Figure 10a–c, the histogram of the original image is unevenly distributed, whereas the histogram in Figure 10d–f, of the encrypted digital Lena image in the encryption transmission system with generalized chaotic synchronization, is uniformly distributed.

### 6.2. Keyspace Analysis

Cryptographic system security and resistance to exhaustive attacks are affected by the size of the key space. The sequences of the 6D discrete hyperchaotic system (21) are applied at the encryption stage, as the production of keys depends mainly on the parameters and initial conditions of the system. Hence, all the iterative variables of the proposed chaotic system ($\begin{array}{cccccccccc} x_{1}, & x_{2}, & x_{3}, & x_{4}, & x_{5}, & x_{6}, & a, & b, & c, & d \end{array}$) can be used as keys. Calculation is performed by means of the double-floating-point number with 64-bit precision; therefore, the key space is $2^{53\times 10}=2^{530}>2^{100}$, which is much large than 2^100^. Consequently, the security of the image encryption system is improved and the resistance to exhaustive attacks is increased [24,25].

### 6.3. Key Sensitivity Analysis

**Figure 11:**
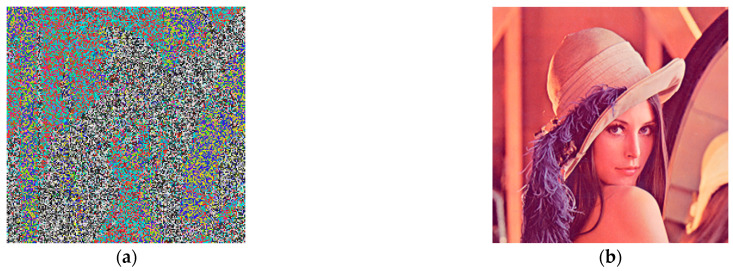
Results of decrypting image using slightly different keystreams: (**a**) decryption result for correct key; (**b**) decryption result for wrong key.

Key sensitivity is an important measure to evaluate the security of an encrypted image. Security is highly reliable if any minimal change to the key results in a large modification; thus, the more sensitive the key, the more secure the encryption system. To evaluate the sensitivity of the key of the proposed scheme, the image was decrypted with a keystream with slight differences, the initial values were set to $\left(x_{1},x_{2},x_{3}\right)=\left(0.02,0.03,0.01\right)$, and the result of making minor changes to the initial values was $\left(x_{1},x_{2},x_{3}\right)=\left(0.02+10^{-15},0.03,0.01\right)$. The results of the experiment are illustrated in Figure 11, in which it can be seen that the image could not be decrypted, even with minor changes to the key.

### 6.4. Cutting Attack Analysis

**Figure 12:**
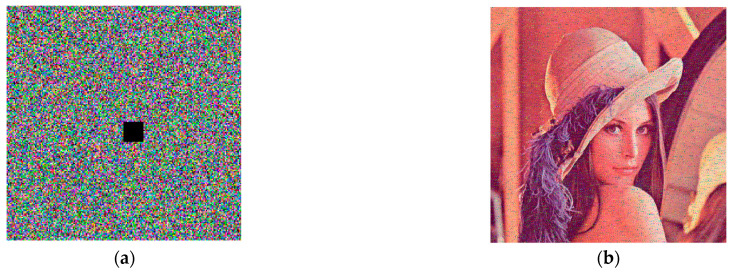
Test result graphs for resistance to data attacks: (**a**) cropped with black pixels in encrypted Lena; (**b**) decrypted Lena.

To prevent data from being attacked or lost, it is necessary to perform cutting attack analysis. The same encrypted Lena as in Section 5.1 was cropped with black pixels, which is shown in Figure 12a. Then, we decrypted the image with keys, and the decrypted image can be well identified, as shown in Figure 12b. Thus the encryption system proposed in this paper is resistant to attacks and data loss as well.

### 6.5. Correlation Analyses

The analysis of image pixel correlation is one of the important indicators of the encryption effect of encrypted images. The color images were analyzed for correlations from R, G, and B, which are represented as red, green, and blue colors, respectively. In addition, the original image has a strong correlation between adjacent pixels, and an effective encryption system can reduce the image pixel correlation considerably. The correlation coefficient results for the Lena test images and their corresponding encrypted images are shown in Table 1. As can be seen from Table 1, the correlation coefficient between adjacent pixels in the original image is close to 1, with a strong correlation, whereas in the encrypted image, neighboring pixels are not correlated, as the correlation coefficient is close to 0.

**Figure 13:**
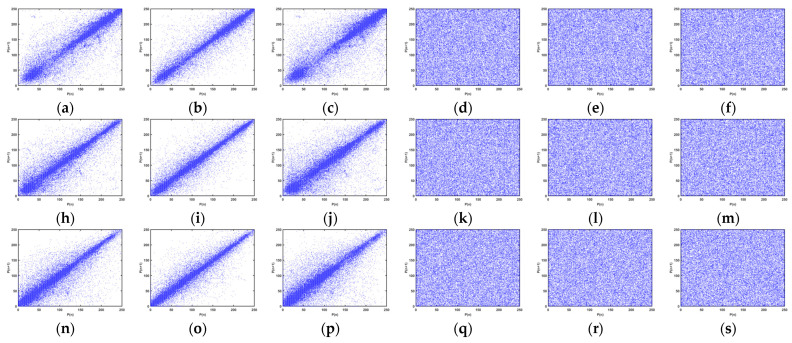
Correlations between pixel points in different orientations of original image: (**a**) correlation between horizontal pixel points of R component; (**b**) correlation between vertical pixel points of R component; (**c**) correlation between diagonal pixel points of R component; (**h**) correlation between horizontal pixel points of G component; (**i**) correlation between vertical pixel points of G component; (**j**) correlation between diagonal pixel points of G component; (**n**) correlation between horizontal pixel points of B component; (**o**) correlation between vertical pixel points of B component; (**p**) correlation between diagonal pixel points of B component. (**d**) correlation between horizontal pixel points of R component; (**e**) correlation between vertical pixel points of R component; (**f**) correlation between diagonal pixel points of R component; (**k**) correlation between horizontal pixel points of G component; (**l**) correlation between vertical pixel points of G component; (**m**) correlation between diagonal pixel points of G component; (**q**) correlation between horizontal pixel points of B component; (**r**) correlation between vertical pixel points of B component; (**s**) correlation between diagonal pixel points of B component.

To demonstrate the correlation visually, we plotted scatter plots of all sampled pixel pairs. The correlations between the test image Lena and its corresponding R, G, and B color pixels are shown separately. Figure 13a–c,h–j,n–p show that the numerical points of the original images are clustered around the diagonal of the images; hence, there is a strong correlation between neighboring pixels about the original image, whereas the values of the points in Figure 13d–f,k–m and Figure 13q–s are evenly spread throughout the entire plane of the images, which indicates that there is virtually no correlation between neighboring pixels in the encrypted image.

## 7. Conclusions

In this paper, two discrete chaotic systems of different dimensions are constructed. Additionally, the dynamics of the new systems are analyzed, and the phase diagram, Lyapunov exponent diagram, and bifurcation diagram of the systems are presented and analyzed simultaneously. The proposed 3D and 6D discrete chaotic systems were constructed as drive systems, and the response systems were constructed by employing the new generalized synchronization method incorporating error-feedback coefficients. The experimental results show that the design of adaptive generalized synchronous systems can be realized provided that the feedback coefficient (η) of the error system satisfies certain conditions for the design of adaptive generalized synchronous systems. Further, the generalized synchronization method incorporating the error-feedback coefficient, and the incorporation of it into the controller, enables simpler and more flexible control of the generalized synchronization. Finally, a chaotic synchronization and encryption–decryption system for secure digital image transmission was constructed by applying the method of generalized synchronous chaotic systems incorporating the error-feedback coefficients devised in this paper. Due to the limited accuracy of the computer, the system proposed in this paper is more resistant to dynamic degradation and, hence, these features of high-dimensional chaotic systems play an active role and have very good theoretical value in image encryption as well as chaotic synchronization.

## Figures and Tables

**Table 1:** Correlation coefficient results for original Lena and encrypted Lena.

Image	Direction	Plain Image			Cipher Image		
		R	G	B	R	G	B
Lena	Horizontal	0.9337	0.9170	0.9088	0.0011	0.0013	−0.0065
	Vertical	0.9669	0.9604	0.9538	0.0068	0.0048	0.0015
	Diagonal	0.9063	0.8886	0.8789	−0.0048	−0.0025	−0.0041

## Data Availability

Not applicable.
